# 

**Published:** 1916-10-21

**Authors:** 


### HOSPITAL AND INSTITUTIONAL RESULTS IN 1915.

Note.—*Medical Superintendents and Secretaries who have not already done so are invited to send a copy of the Annual Report for review in this column.*

**Royal Earlswood Institution.** — The report complains of depleted income and indebtedness to bankers and tradesmen to the extent of £9,000. The accounts are not kept under the Uniform System, and are difficult to follow; the income for the year appears to have been £31,184, and the expenditure £35,357. There was a loss of £144 in annual subscriptions. There were 160 mentally defective children on the school register at the close of the year.

**James Murray's Royal Asylum, Perth.** — The average cost per patient for the year was £105. The income amounted to £16,147, of which £13,791 was received in payments for board; the expenditure was £14,512. The report of the Commissioners of the General Board of Control is of a satisfactory nature.

**Hull City and County Asylum.**—Owing to Treasury restrictions affecting capital expenditure the building of the two detached villa blocks has been suspended. The farm accounts showed a profit of £425. The maintenance rate of Poor-Law patients has been increased from 11s. 8d. per week to 13s. The cost of printing the report, with its many elaborate tables, must have been considerable; we do not see the utility of recording the cash emoluments of each individual member of the staff.

**Wilts County Asylum.**—There were 1,108 persons in the institution at the close of the year. The death-rate for 1915 was 8.6, the average death-rate for public asylums throughout England and Wales being 10. A new block was in process of erection, but work was stopped in December last owing to the refusal of the Treasury to sanction any further loan. The dinner on the day of the visit of the Commissioner of the Board of Control was pea-soup, with bread and cheese and coffee.

**Hants County Asylum, Fareham.**—At the close of the year there were 1,317 patients in the asylum, the sexes being nearly equally divided. The percentage of recoveries to admissions is 21.9 males, 32.2 females. The cost of maintenance was slightly over 10s. per week for the year ending March 31, 1915. The Commissioners of the Board of Control report favourably upon the administration of the institution and the care of the patients.

### Hospitals at Lyons and Limoges.

The two military hospitals maintained in France, at Lyons and Limoges respectively, by the Wounded Allies Relief Committee, for French wounded soldiers, are doing most useful work, and the report of Sir Lindsey Smith, honorary secretary, who has recently been over to France to inspect the hospitals, is altogether most satisfactory. M. Godart, Ministre de Santé, and General Collin expressed to Sir Lindsey Smith their warm approval of the "Entente" hospitals. "It is interesting to know that, although many of our nurses can only speak the language of the wounded very slightly, the latter are, nevertheless, quite happy to be in our English hospitals," said Sir Lindsey, in speaking of his visit of inspection. Of the two hospitals, the one at Limoges has recently received the greater number of wounded. This is largely due to the fact that the Lyons Hospital is on the heights some distance from the town, and when the wounded arrive by train only as many patients are received as can be fetched in one journey by the ambulances, the number of which is naturally limited. The Lyons Hospital contains 300 beds; Dr. Simpson and Miss Walker, M.B., are the residents, and Miss Watson, late assistant matron of St. George's Hospital, is the matron.

### Matrons' and Sisters' Appointments.

BLENCATHRA SANATORIUM, THRELKELD.—Miss J. F. Gillespie has been appointed matron. She was trained at Glasgow Eastern District Hospital, where she was later sister, and has since been sister at the Grampian Sanatorium, Kingussie, and at Dousland Sanatorium for Women, and matron of the Udal Torre Sanatorium for Men.

GOLDIE LEIGH HOMES.—Miss S. Hellesen has been appointed by the Metropolitan Asylums Board acting matron of the Goldie Leigh Homes. She was trained at the Sussex County Hospital, Brighton, and was appointed assistant matron of the High Wood School (feeble-minded colony) in 1908, since which time she had been continuously in the service of the Metropolitan Asylums Board as home sister and assistant matron of the Downs School, and supernumerary home sister at the Park Hospital for Children. She has acted as matron of the Goldie Leigh Homes since the death of the late matron last November.

RIVER HOSPITALS AND AMBULANCE SERVICE.—Miss N. H. Thorpe, first assistant matron at the Brook Hospital, has been appointed by the Metropolitan Asylums Board matron to the River Hospitals and Ambulance Service in succession to Miss Wacher, who has resigned. Miss Thorpe was trained at Croydon Infirmary, and has been in the Board's service upwards of twenty-three years, first as charge nurse at the North-Eastern Hospital, and later at the Brook Hospital as charge nurse, night superintendent, and assistant matron.

BELBROUGHTON, BOURNE CASTLE SANATORIUM.—Miss G. M. Mackenzie has been appointed sister. She was trained at St. Marylebone Infirmary, and has since been a sister of Queen Alexandra's Imperial Military Nursing Service Reserve.

BLACKBURN FEVER HOSPITAL.—Miss E. M. Bramhill has been appointed night sister. She was trained at Gloucester Royal Infirmary and Monsall Fever Hospital, Manchester, and has since been staff and charge nurse at Lincoln City Hospital, and temporary night sister at Blackpool County Borough Hospital.

CARDIFF WORKHOUSE INFIRMARY.—Miss Rose Leary has been appointed night sister. She was trained at Dudley Road Infirmary, Birmingham, where she has since been night superintendent.

**NOTE.—Particulars of Appointments for insertion in this column will be welcomed.**

### Obituary.

THE death has occurred at Hull, at the age of seventy-three, of Mr. George Lamb, honorary surgeon of the Hull Royal Infirmary and deputy-surgeon at the prison.

IT is with regret we have to announce the death from wounds received in action of Mr. A. R. Dyball, who for two and a-half years prior to the outbreak of war held the appointment of clerk in the secretary-superintendent's office at Addenbrooke's Hospital, and has been serving his King and country for the past two years. By his sterling qualities and great devotion to the best interests of the hospital, Mr. Dyball endeared himself to all with whom he came into contact. Expressions of deep sympathy have been conveyed to his family by the committee, the secretary-superintendent, by Mr. H. F. J. Mann, chief clerk and accountant, and other members of the staff.

RATES OF SUBSCRIPTION: Twelve months. 6/6; Six months, 4/-; Three months, 2/-; post free to any address in the United Kingdom. Abroad, Twelve months, 8/8; Six months, 5/-; Three months, 2/6, post free. THE HOSPITAL "Index" to Volume LX., April to September 1916, is now ready, price 1½d. per copy, post free. Address The Manager, "THE HOSPITAL," The Hospital Building, 28/29 Southampton Street, Strand, London, W.C.

